# Application of VCSEL in Bio-Sensing Atomic Magnetometers

**DOI:** 10.3390/bios12121098

**Published:** 2022-11-30

**Authors:** Peng Zhou, Wei Quan, Kai Wei, Zihua Liang, Jinsheng Hu, Lu Liu, Gen Hu, Ankang Wang, Mao Ye

**Affiliations:** 1 School of Instrumentation and Optoelectronic Engineering, Beihang University, Beijing 100191, China; 2 Beihang Hangzhou Innovation Institute Yuhang, Xixi Octagon City, Yuhang District, Hangzhou 310023, China

**Keywords:** VCSEL, chip-scale atomic magnetometers, magnetoencephalography, magnetocardiography

## Abstract

Recent years have seen rapid development of chip-scale atomic devices due to their great potential in the field of biomedical imaging, namely chip-scale atomic magnetometers that enable high resolution magnetocardiography (MCG) and magnetoencephalography (MEG). For atomic devices of this kind, vertical cavity surface emitting lasers (VCSELs) have become the most crucial components as integrated pumping sources, which are attracting growing interest. In this paper, the application of VCSELs in chip-scale atomic devices are reviewed, where VCSELs are integrated in various atomic bio-sensing devices with different operating environments. Secondly, the mode and polarization control of VCSELs in the specific applications are reviewed with their pros and cons discussed. In addition, various packaging of VCSEL based on different atomic devices in pursuit of miniaturization and precision measurement are reviewed and discussed. Finally, the VCSEL-based chip-scale atomic magnetometers utilized for cardiac and brain magnetometry are reviewed in detail. Nowadays, biosensors with chip integration, low power consumption, and high sensitivity are undergoing rapid industrialization, due to the growing market of medical instrumentation and portable health monitoring. It is promising that VCSEL-integrated chip-scale atomic biosensors as featured applications of this kind may experience extensive development in the near future.

## 1. Introduction

Nowadays, magnetocardiography (MCG) is attracting growing interest due to outstanding performance on screening and diagnosis of heart disease. Compared with the traditional electrocardiogram (ECG), multi-channel magnetocardiographic mapping (MMCG) is a faster and contactless method for 3D imaging and localization of cardiac electrophysiologic phenomena with higher spatial and temporal resolution [1]. In addition to MCG, magnetoencephalography (MEG) emerges as an important approach for the characterization of functionality and effective connectivity of the brain and has already been applied for the research of cognitive science [2,3]. In conventional practice, the superconducting quantum interference device (SQUID) is considered as a feasible approach of neuromagnetic fields, namely brain and heart [4]. However, SQUID systems is expensive and cumbersome as it requires liquid Helium circulation for temperature conditioning, which has made it challenging to be applied to common research and clinical sites [5]. On the other hand, the emerging atomic magnetometer has the same level of sensitivity with much lower cost and volume, which has long been considered as a feasible substitute of SQUID. Driven by the urgent demand, the atomic magnetometer (AM) has experienced dramatical development in the past two decades accompanied by the development of nano fabrication technologies. Researchers nowadays are able to utilize AM as an alternative (of SQUID) for clinical applications. One of the major trends for AM is the volume reduction that facilitates the integration of multiple sensors in a small area in pursuit of high imaging resolution—especially chip-scale atomic magnetometers that enable integration of sensor arrays to obtain three-dimensional magnetic field signals with flexible signal localization and fast screening [6,7,8,9]. However, due to the requirement of chip integration, conventional bulk lasers are not suitable for emerging chip-scale atomic magnetometers. On the other hand, VCSEL have advantages including low power consumption, feasible digital modulation, circular beam profiles, and compact volume, which are promising characteristics for the realization of chip-scale atomic magnetometers.

In this review, we summarize the application of VCSELs in chip-scale atomic devices, focusing on chip-scale atomic magnetometers, where VCSELs are integrated in various bio-sensing atomic sensors. The VCSEL designed for atomic transition (pumping) wavelength is summarized. Then, the mode and polarization control methods of VCSELs in pursuit of sensor sensitivity and stability. Finally, the application of chip-scale magnetometer in the field of cardiac and brain magnetometry is discussed in detail. Nowadays, the market of high-spatial-resolution medical imaging and portable medical instruments is undergoing rapid expansion, pushing the demand for VCSELs (of alkali-metal pumping wavelengths) integrated chip-scale bio-sensors to an unprecedented level. It is promising that VCSEL-integrated chip-scale atomic bio-sensors and related technologies may experience extensive development in the near future.

## 2. The Fundamentals of VCSEL and Atomic Magnetometer

### 2.1. VCSEL Fundamentals

Nowadays, the VCSEL is widely acknowledged as a commercialized light source for emerging applications, namely integrated light detection and ranging (LIDAR) and Virtual Reality/Augmented Reality (VR/AR). While the wavelength needed for atomic devices is normally fixed by transition band gap of alkali atoms, namely 795 nm(Rb), 895 nm(Cs). However, VCSEL for these wavelengths have not been fully commercialized, especially high power VCSEL over tens of mW (at atomic transition wavelengths). Many efforts have been made to develop VCSELs and the integration into atomic devices in recent years. In this chapter, the history and fundamental structure of VCSEL are briefly reviewed.

VCSEL, since it first came out in 1977, has experienced dramatic development in recent decades. In 1977, Kenichi Iga first proposed the concept of a vertical cavity surface emitting laser and successfully made the world’s first VCSEL in 1979 [10], which can only work at ultra-low temperatures. Later, with the advancement of nanofabrication technologies, researchers made improvements to the structure of VCSELs. The first is the growth of distributed Bragg reflectors using molecular beam epitaxy (MBE) or metal organic vapor chemical deposition (MOCVD) [11,12,13,14]. The second is the use of a quantum well structure in the active region, which effectively reduces the threshold current of the VCSEL and makes it possible for the VCSEL to operate at room temperature [15,16,17,18,19,20]. The third one is the proposed oxidation aperture technique, which can be limited to both optical and electric fields [21,22,23]. Meanwhile, even better performance can be obtained by slight modification to the VCSEL structure, such as surface relief [24,25,26] and high-index-contrast subwavelength grating [27,28,29].

**Figure 1:**
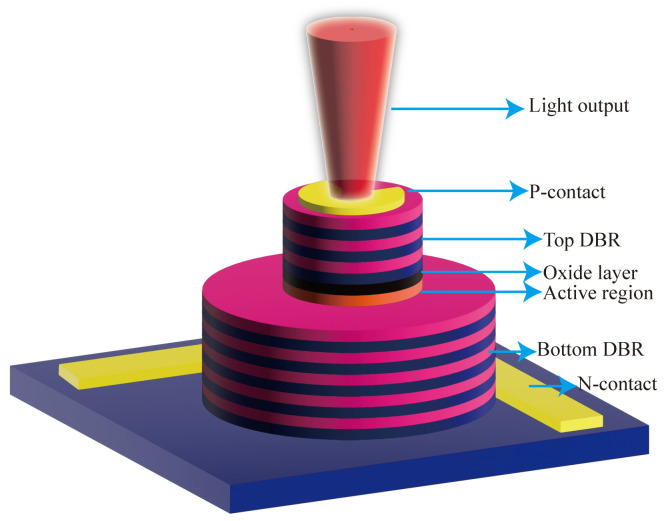
Structure of VCSEL.

The VCSEL is composed of an optical cavity with a two distributed Bragg reflector (DBR) and a gain region. The top and bottom mirrors are periodic structures consisting of high and low refractive index materials of quarter wavelength, and their reflectivity should generally exceed 99%. By changing the number of periods of the high and low refractive index materials of the top and bottom mirrors, it is possible to achieve either top-emission or bottom-emission of the VCSEL. The intermediate active region is usually composed of one or several quantum well structures that are only a few tens of nanometers thick. VCSELs with different emission wavelengths can be obtained by designing active region materials with different components. For 795 nm (Rb) and 894.6 nm (Cs), the active materials are usually composed of $In_{x}AlGa_{1-x}As$ or $Al_{x}Ga_{1-x}As$. To precisely control the thickness of each layer of the structure, DBR is generally grown by MOCVD or MBE. The top and bottom of the VCSEL need to be coated with a ring-shaped metal layer to allow the current to be injected through the ohmic contact. Substrates usually use a p-on-n doping sequence to reduce absorption loss of n-type mirrors. To increase the current limitation and light field limitation of VCSELs, oxide apertures are formed by selectively lateral oxidizing a 10 nm–30 nm thick semiconductor layer with high aluminum content. The material of the oxide layer is typically AlAs. Figure 1 shows the schematic structure of the VCSEL. The current is injected from positive electrode into the active region through oxide aperture. When the condition of stimulated emission is satisfied, the photons are reflected by DBRs and form a standing wave in the resonant cavity. As the stimulated emission continues to intensify, the laser is emitted from the DBR where the reflectivity is smaller. Consequently, VCSELs emit in a single transverse-mode with an approximately Gaussian shape by selective lateral oxidation.

### 2.2. Atomic Magnetometer

**Figure 2:**
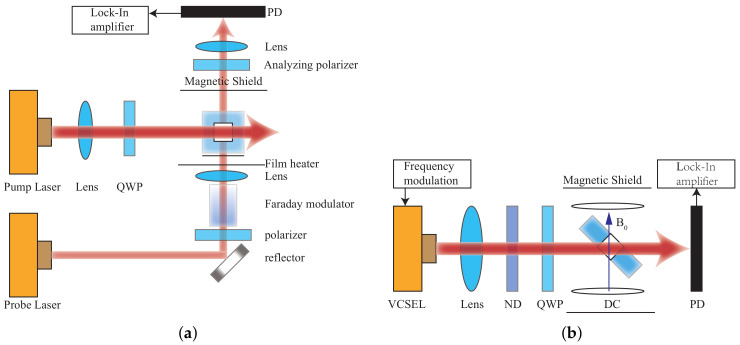
(**a**) Structure of SERF magnetometer; (**b**) structure of the Bell–Bloom type SERF magnetometer.

**Figure 3:**
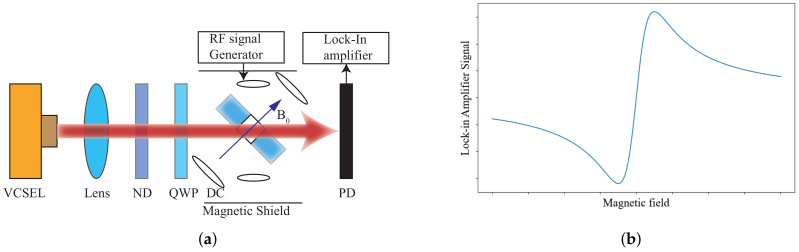
(**a**) Structure of M-x magnetometers; (**b**) dispersive signal as a function of the magnetic field.

With the increase of sensitivity to the order of femtotesla, chip-scale atomic magnetometers have been used for cardiac and cerebral magnetic measurements. Generally, the magnetometer relies on the Zeeman splitting at the atomic level, not the hyperfine level splitting [30]. Chip-scale atomic magnetometers suitable for miniaturization are based on optical pumping. When $\sigma^{+}-$ polarized light in resonance with the D1 line pass through the alkali metal vapor, the alkali metal atoms in the ground state are pumped to the excited state and made to become spin-polarized along the direction of the pumped light. Consequently, the atoms are pumped into the optically non-absorbing dark state using $\sigma^{+}-$ polarized laser beam, and then the oscillation frequency of the magnetic field and the Zeeman splitting resonance is set to drive the atoms from the dark state into the absorbing state, so that the magnetic resonance jump can be detected by changes in transmitted light through cell [31]. Atomic magnetometers can be divided into many categories; here, we focus on three types of widely used chip-scale atomic magnetometers. The first is, the chip-scale atomic magnetometer is based on the CPT resonance principle, which probes the ground-state hyperfine splitting between two magnetically sensitive Zeeman states to measure the magnetic flux density experienced by an atom ensemble. The second one is a chip-scale atomic magnetometer based on the spin–exchange relaxation-free (SERF) principle. Atoms with high alkali metal atomic densities and very low magnetic fields have Larmor frequencies much smaller than the relaxation rate of the atoms. When a $\sigma^{+}-$ polarized pump beam of a specific frequency passes through alkali vapor cell, the spin direction of the atomic ensemble is redirected. If there is a weak magnetic field perpendicular to the direction of polarization, the polarization of the atoms will be deflected. There are two main ways to detect this deflection signal. One is based on the Faraday effect, where a linearly polarized light is applied perpendicular to the direction of atomic polarization, and the polarization plane is deflected when the line polarized light passes through the alkali vapor cell [32]. The structure of the dual-beam SERF magnetometer is shown in Figure 2a. Another way is to frequency modulate or amplitude modulate the pump light. The process of atomic polarization reorientation leads to an increase of light absorption in the alkali vapor cell, so that the transmission spectrum of light as a function of the magnetic field will have a zero-field resonance. The measured absorption curve is transformed into a dispersion curve by a lock-in amplifier, at which point there is a maximum slope suitable for detection at the zero fields. The structure of optical magnetometry with frequency modulated is shown in Figure 2b. In addition to light modulation, magnetic field modulation methods can also perform magnetic field measurements [32]. The structure of the M-x magnetometer is shown in Figure 3a, and the schematic diagram of the dispersion curve obtained by the lock-in amplifier is shown in Figure 3b.

The above description of the atomic device principle shows that the basic requirement for a VCSEL is a single frequency output, which means that the VCSEL must be single mode and single polarized. Maintaining single mode and single polarization suppresses laser phase noise and intensity noise, which can narrow the laser linewidth and increase instrument accuracy. For VCSEL applications in atomic devices, the line width of the laser should be less than 100 MHz [33]. Next, I will introduce the development of VCSEL mode control and polarization control, respectively.

## 3. Mode Control of VCSEL for the Chip-Scale Atomic Magnetometer

In the chip-scale atomic magnetometer, the transverse higher-order modes affect the polarization rate of alkali metal atoms and reduce the accuracy of chip-scale atomic devices. Therefore, it is necessary to ensure the single transverse mode output of the laser. Generally, the VCSEL can be approximated as a cylindrical waveguide structure, which provides transverse optical confinement. The cavity length of the VCSEL is only one or a few wavelengths, and it is generally a single longitudinal mode output. However, its transverse dimension is larger than its effective cavity length, and higher-order modes often appear in the transverse direction. There are many ways to achieve single-mode VCSELs, such as using external mirrors for mode control, which results in a VCSEL with high single-mode power. However, we need to reduce the size of the atomic devices, so the external mirror approach is avoided as much as possible. The next main consideration is the method of achieving single-mode output from inside the VCSEL. Generally, there are two main categories to achieve single-mode output from the VCSEL itself: transverse optical guiding and employing mode-selective losses or gain [34].

### 3.1. Transverse Optical Guiding

There are many ways to realize lateral optical guiding, and the simplest way is to reduce the oxide aperture diameter. In 1997, Grabherr et al. realized an 850 nm VCSEL with a single-mode output power of 2.8 mW at a 3 μm oxide aperture [35]. In the same year, Jung et al. realized an 840 nm VCSEL with a single-mode power output of 4.8 mW at a 3.5 μm oxide aperture [36]. However, the smaller oxide aperture increases the thermal resistance of the VCSEL, leading to an increase in the self-heating effect of the VCSEL. The high temperature causes changes in VCSEL output power and threshold current, shortening the lifetime of the VCSEL.

**Figure 4:**
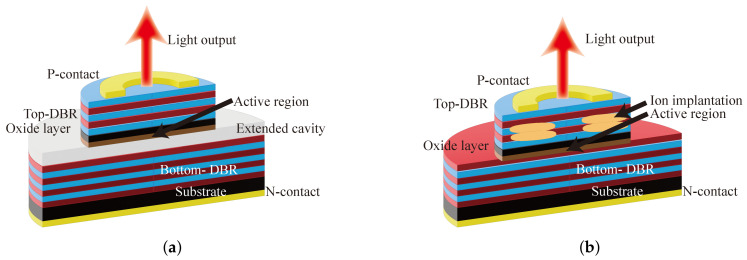
Structure of the different methods for realizing single-mode outputs (**a**) Extended cavity VCSEL; (**b**) ion implantation VCSEL.

To solve the problem of serious heat generation in small oxide aperture VCSELs, the extended cavity technique is proposed. Figure 4a illustrates the structure of the extended cavity VCSEL. The diffraction loss increases with increasing the length of a resonant cavity. The higher-order mode has a larger lateral range with respect to the fundamental modes, and their losses are greater. By using the extended cavity technique, a single mode VCSEL has been achieved. In 2000, Unold et al. demonstrated a single mode VCSEL with long monolithic cavity, whose output power reached 5 mW at 7 μm oxide aperture [37]. Later, Unold et al. further realized a single mode output power of 5.4 mW at 980 nm emission, which had an effective cavity length of 9.2 μm [24]. The extended cavity VCSEL increases the cavity length, which may lead to an increase in the longitudinal modes. Moreover, increasing the cavity length will attenuate the fundamental mode while attenuating the higher-order modes, reducing the output power of the VCSEL.

A combination of ion implantation and selective oxidation techniques can also be used to achieve single-mode VCSELs, setting the aperture diameter formed by ion implantation slightly smaller than the oxidation aperture diameter. Figure 4b illustrates the structure of the ion implantation VCSEL. This approach limits both the optical and electric fields, concentrating the current in the central region through a smaller ion implantation diameter, increasing the gain to the fundamental mode while increasing the loss to higher-order modes. In 2001, E.W. Young et al. achieved a peak output power of 4.5 mW and an SMSR of 45 dB in an 850 nm VCSEL with an oxide aperture of $9\times 9\;\mu m^{2}$ and an ion implantation aperture of 6 μm [38]. In 2004, an 850 nm VCSEL fabricated by Fang-I Lai et al. achieved an output power of 3.8 mW with an oxide aperture of 8 μm [39]. The Zn-diffusion approach is similar to the ion implantation approach in that it achieves single mode output by increasing the optical loss in higher modes, but the deep Zn-diffusion layer increases the threshold current and reduces the output power. In 2001, C.C. Chen et al. solved the problem by a shallow Zn-diffusion technique [40]. Later, J.W. Shi et al. used the Zn-diffusion technique to enhance the single-mode VCSEL to obtain a bandwidth of 8 GHz and a maximum output power of 3 mW [41]. In 2013, J.W. Shi et al. integrated a single-mode VCSEL with Zn-diffusion technique into a 6 × 6 two-dimensional array with a dispersion angle of 4° and an output power of 104 mW [42]. The top DBR deposited metal layer to form a metal aperture is a further improvement of the above structure, and the metal aperture is slightly larger than the oxidation aperture, the lateral range of the higher order die is larger and more easily blocked by the metal layer, the base die is mainly in the central region, and the blocking effect is weak. In 2006, Otoma et al. fabricated an 850 nm single-mode VCSEL with an output power of 4.7 mW and an SMSR of 27 dB using a structure limited by metal aperture and oxide-confined [43].

### 3.2. Mode-Selective Losses or Gain

**Figure 5:**
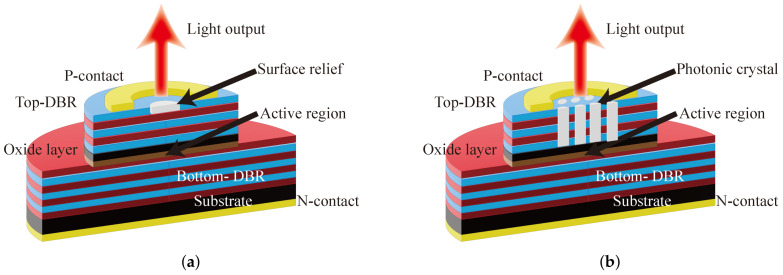
Different structures to realize single-mode outputs (**a**) surface relief VCSEL; (**b**) photonic crystal VCSEL.

The single-mode output can be achieved by keeping the loss in the center region constant through surface relief. The reflectivity of the DBR structure is very sensitive to changes in the number of layers of high and low reflectivity as well as changes in layer thickness; therefore, the loss in the high-order mode region can be increased by surface relief while the loss in the fundamental mode part of the central region is kept constant to achieve single-mode output. In 1999, H.J. Unold et al. first proposed a surface relief technique for mode control and achieved a single-mode output VCSEL with a wavelength of 850 nm at μm aperture, which has an output power of 2 mW and an SMSR of 30 dB [44]. The profile of surface relief VCSEL is shown in Figure 5a. In 2001, H.J. Unold et al. proposed a surface relief self-alignment method, which requires only one additional photoresist step, and achieves single-mode output at 16 μm oxide aperture, which has an output power of 3.4 mW at room temperature [24]. In 2009, Pierluigi Debernardi et al. established a hot-cavity model for surface relief VCSELs and standard VCSELs and found that the thermal lensing effect of surface relief structure is more obvious [26]. Surface relief VCSELs are single-mode outputs at the same drive current, while standard VCSELs are multimode outputs. Compared to other methods, the surface relief technique causes little damage to electrical or thermal characteristics. However, the surface etching technique requires very high precision etching accuracy, while it is not easy to batch production. An inverted surface relief technique can overcome the drawback. In 2016, Benjamin Kesler et al. proposed the method of depositing patterned dielectric layers on VCSELs, which has the advantage that it can be applied to any VCSEL without etching or epitaxial growth process, and the output power is 3.5 mW at 850 nm wavelength [45]. In 2017, for the application of VCSELs in CPT atomic clocks, Lei Xiang et al. performed surface relief etching in the center of the top reflector to implement a single-mode VCSEL [46]. The device uses gain cavity detuning to solve the emission wavelength shift problem. The single-mode output power reaches 0.45 mW at 80 °C, while the side mode rejection ratio exceeds 30 dB. In atomic devices, large output power is usually one of the requirements for a laser. In 2018, Zuhaib Khan combined surface etching and Zn diffusion layer techniques for high-speed VCSELs to increase the output power while reducing the number of modes to achieve quasi-single-mode output [47].

Another effective method for VCSELs to achieve single-mode output is to form a two-dimensional photonic crystal structure by etching the top DBR. The profile of photonic crystal VCSEL is shown in Figure 5b. A single-mode cylindrical waveguide is formed in the central region by varying the depth, shape, and spacing of the etched holes. In 2002, Dae-Sung Song et al. fabricated an 850 nm photonic crystal VCSEL with an SMSR of 45 dB, modeled after the structure of a photonic crystal fiber [48]. However, photonic crystal structure increases optical losses and thermal resistance, resulting in a large threshold current and low energy efficiency. Danner and Yang made improvements for photonic crystal power enhancement, achieving an output power of 3.1 mW at 850 nm and 5.7 mW at 990 nm, respectively [49]. In 2013, Meng Peun Tan et al. combined ion-implanted structures and photonic crystals to reduce the series resistance and current density of VCSELs by separating electrical and optical apertures, with an output power of more than 2.5 mW at 850 nm [50].

**Figure 6:**
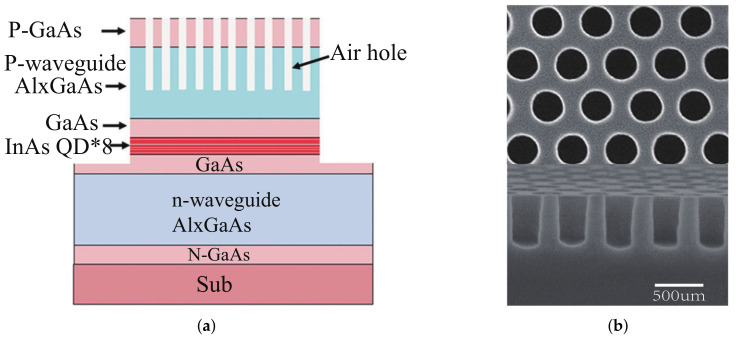
(**a**) Cross-sectional schematic of the PCSEL; (**b**) top-view SEM image of the 2D PC and side-view SEM image of the 2D PC [53].

VCSELs generate high order modes at higher currents due to the complex doping of DBR, oxide hole limiting structure, and other factors. The previously adopted method is to integrate photonic crystals into the DBR, and this structure still suffers from multi-mode problems at high currents. Directly replacing the DBR with a two-dimensional photonic crystal can obtain a single fundamental mode output at higher currents. Photonic crystal surface emission lasers (PCSELs) are used to achieve large range surface laser emission through multi-directional Bragg diffraction of photonic crystals, while the beam direction and polarization characteristics of the laser can be easily controlled. In 2014, Kazuyoshi’s work led to a breakthrough in photonic crystal SEL power enhancement by using the MOCVD method instead of wafer bonding to construct a triangular hole to form a photonic crystal structure with a single-mode output power of 0.5 W [51]. In 2017, Ming-Yang Hsu et al. achieved the first 1.3 μm quantum dot photonic crystal surface emission laser(QDPCSEL) by using indium tin oxide (ITO) deposition instead of the sacrificial layer etching technique and epitaxial regrowth technique or wafer bonding technique, simplifying the process flow while obtaining a better current uniformity and finally achieving an output power of 2 mW [52]. In 2019, Huan-Yu Lu et al. designed QDPCSEL with an output power of 13.3 mW at a high temperature of 90 °C and a wavelength of 1.3 μm by introducing an additional lateral feedback mechanism [53]. The cross-sectional views of PCSEL and SEM image of photonic crystal are shown in Figure 6a and Figure 6b, respectively. In 2021, Lih-Ren Chen et al. also used P-surface ITO deposition to improve the output efficiency of the laser, comparing the slope efficiency of different air hole shapes, and found that increasing the asymmetry of the air hole shape could improve the slope efficiency of the laser [54].

**Figure 7:**
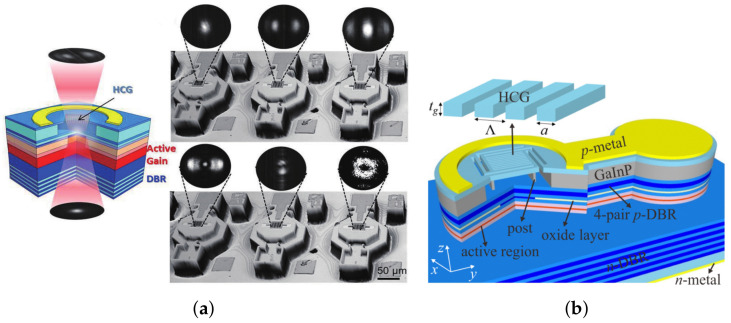
(**a**) Double-sided VCSEL structure and its beam shaping effect [58]; (**b**) schematics of the 940 nm HCG-VCSEL [57].

The high-contrast subwavelength grating (HCG) structure provides a new implementation idea for the single-mode output of VCSELs. The HCG structure is a periodic structure consisting of a high refractive index grating strip and a low refractive index medium with a grating period between the high and low refractive index wavelengths. The output light mode and reflectivity can be easily controlled by changing the grating period, thickness, duty cycle, and other parameters. In 2007, Michael C.Y. HUANG et al. replaced the HCG structure with a partial VCSEL top DBR, achieving an output power of about 1 mW at room temperature with an SMSR of up to 45 dB [55]. HCG has a lower thermal resistance compared to DBR and can achieve a higher reflectivity in a smaller volume. Therefore, it is well suited for single-mode output at large oxide apertures. In 2008, Michael C.Y. HUANG et al. compared the output power of VCSEL with different grating areas to investigate the single-mode characteristics of HCG-VCSEL with an output power of 2.3 mW at 10 μm oxide aperture and its SMSR over 40 dB [56]. Because of the characteristics of HCG structure such as high emissivity and easy tuning, long wavelength single mode VCSELs come to be used for optical communication. In 2010, Werner Hofmann et al. using amorphous silicon to fabricate HCG, achieved the first electrically pumped VCSEL at 1310 nm using an HCG structure and obtained single-mode emission with an output power of more than 0.4 mW at an oxide aperture of 11 μm [27]. In 2013, Y. Rao et al. realized an InPd-based 1550 nm VCSEL with 2.4 mW single-mode output at 15 °C continuous wave operation [29]. In 2018, KunLi et al. constructed a novel beam shaping element using HCG to realize a double-sided VCSEL with different mode distributions, where one side has a single-mode output and the other side has a different far-field emission mode. Figure 7a shows its double-sided emission structure and beam shaping effect. In 2022, Jing Zhang et al. fabricated an electrically injected VCSEL with a rear-supported HCG structure and a laser with single-mode output in the 0–2 mA range and an SMSR of 43.6 dB at 25 °C continuous-wave operation [57]. Figure 7b shows the schematics of the 940 nm HCG-VCSEL. Research progress of single-mode control method of VCSEL is shown in Table 1.

## 4. Polarization Control of VCSEL for Chip-Scale Atomic Device

In chip-scale atomic sensors, optical pumping requires a polarization-stabilized light source. However, the structure of the VCSEL has cylindrical symmetry, the top DBR is not selective for the polarization direction of the VCSEL, and the birefringence caused by the stress–strain introduced during the fabrication process makes the polarization direction of the light emitted from the VCSEL grown on the (001) substrate indeterminate along the (110) or (1–10) direction out. The output polarization of the VCSEL is inherently unstable. In addition, for VCSELs with a specific polarization mode, a sudden change in polarization mode occurs by changing the laser injection current or external perturbations (substrate temperature, chip heat source, optical feedback, etc.), which is called polarization switching. In general, the physical mechanisms affecting the polarization characteristics of VCSELs are so diverse that stable polarization cannot be achieved by solving them individually. Therefore, the basic principle of polarization stabilization is to enhance the polarization of our choice and suppress other adverse effects to ensure that this polarization dominates. According to this principle, polarization stabilization can be achieved by changing the polarization-related gain or loss.

### 4.1. Anisotropic Loss

**Figure 8:**
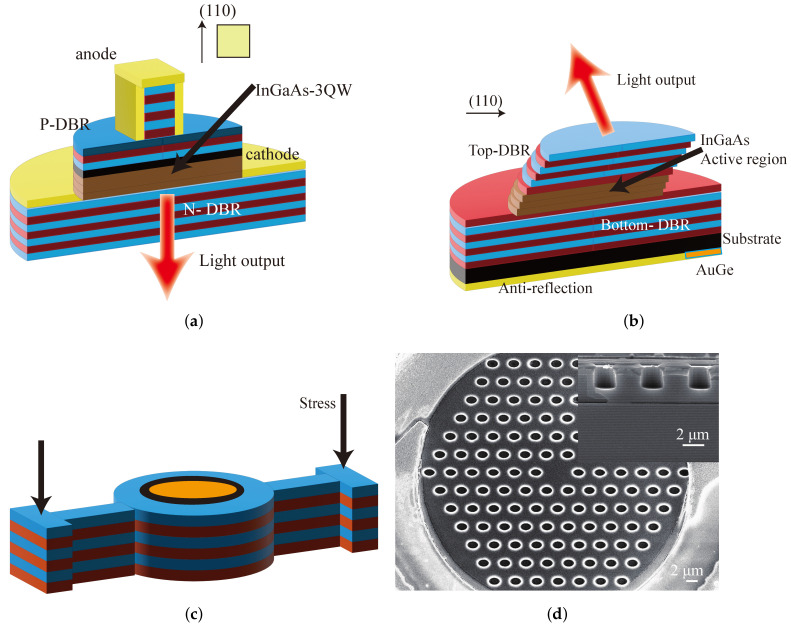
Anisotropic loss VCSEL (**a**) schematic cross-sectional layout of the VCSEL with rectangular column [64]; (**b**) schematic cross-sectional layout of the VCSEL with tilt column [67]; (**c**) schematic drawing of the VCSEL with the oxidized “T-bars” [68]; (**d**) SEM images of the fabricated photonic crystal VCSEL [71].

The introduction of small non-uniform losses can stabilize the polarization direction, where the simplest way is to construct an asymmetric table structure. In 1998, Takashi Yoshikawa changed the VCSEL columnar table structure to a rectangular structure with the short side of the rectangle parallel to the (110) direction, and polarization control is achieved by shortening the length of the short side to change the loss [64]. Figure 8a illustrates this structure. In addition to the rectangular table structures, there are also dumbbell structures [65] or oval structures [66]. The advantage of this approach is that the output power and beam quality are less affected. Another way to introduce polarization loss is to construct a slanted column structure by etching the substrate at an angle of 15–30 degrees in the (110) or (1-10) direction using reactive beam ion etching to etch the cylindrical table structure. Figure 8b illustrates this structure. The VCSEL main polarization direction is perpendicular to the laser column, and the final achieved orthogonal polarization rejection ratio(OPSR) is 25 dB [67].The stress T-bar structure is also used for polarization control, and its structure is shown in Figure 8c. This structure reduces the probability of polarization flip from 32% to 2% compared to the circularly symmetric structure [68]. In 2022, Yen-Yu Huang et al. achieved polarization control by strain introduced by plating the copper substrate on the back of the VCSEL, which was experimentally shown to enhance OPSR without degrading slope efficiency [69]. Another way to introduce non-uniform loss is to etch elliptical holes on the top DBR of the VCSEL, which not only ensures that the VCSEL is emitted in a single transverse mode but also stabilizes the polarization direction of the VCSEL, as Figure 8d shows.

In 2003, Dae-Sung Song et al. achieved polarization control by introducing an elliptical hole in the top DBR, where the polarization control is achieved by the asymmetric structure formed by the elliptical hole and the asymmetric current injection due to the different centering of the hole diameter from the ring electrode formed by proton injection. The OPSR of VCSEL reaches 29 dB [70]. In 2015, Yiyang Xie et al. used the finite difference time domain method (FDTD) method to analyze the effect of different parameters of elliptical photonic crystals on polarization control, and the fabricated VCSEL finally achieved an output power of 1.6 mW and an OPSR of 20 dB [71]. The disadvantage of the photonic crystal structure is that it destroys the thermal and electrical properties of the original VCSEL structure. In 2016, C.M. Long et al. introduced birefringence and dichroism by etching two symmetrically aligned arcs above the gain structure in the laser cavity to enhance the polarization control of the VCSEL [72]. In 2018, Jiye Zhang used an eye-type oxide aperture for polarization control with an OPSR of 22 dB [73]. In 2021, Jiye Zhang et al. constructed anisotropic oxidation apertures through asymmetric airflow distribution during wet oxidation, ultimately achieving an SMSR of over 20 dB and a side mode rejection ratio of over 25 dB [57]. The disadvantage of this method is that the asymmetric oxidation aperture leads to an asymmetric far-field distribution.

### 4.2. Anisotropic Gain

**Figure 9:**
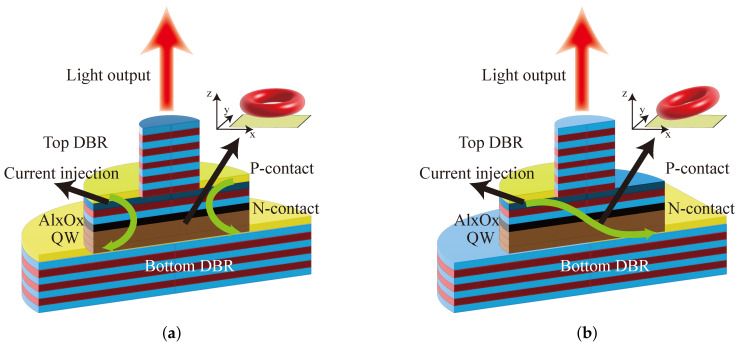
(**a**) VCSEL with symmetric current injection; (**b**) VCSEL with asymmetric current injection.

The choice of active region is mainly determined by the wavelength and substrate. However, VCSELs with AlGaAs as the active region cannot meet the wavelength requirements of atomic sensing. The quantum dots (QD) structure in the active region of VCSEL has to be adjusted in a way that the bandgap energy of QDs fits to the new emission wavelength. For that purpose, indium was introduced into the AlGaAs material system used for the QDs of the VCSELs. In addition to the introduction of non-uniform losses, asymmetric polarization dependent gain can also be used for polarization control. In 1997, Hideaki Saito et al. constructed anisotropic quantum dot structures to control the polarization of VCSELs by varying the intensity of light along two mutually perpendicular directions, achieving an OPSR of 18 dB for VCSELs [74]. In 1998, Akimasa Mizutani realized an oxide-limited InGaAs-GaAs VCSEL on GaAs(311)B substrate with an ultra-low threshold current and an orthogonal polarization rejection ratio of over 30 dB [75]. In 1999, Akimasa Mizutani et al. further investigated VCSELs on GaAs(311)B substrates, which exhibited stable single transverse mode and polarization at 5 GHz under sinusoidal modulation with SMSR and OPSR exceeding 30 dB and 10 dB, respectively [76]. However, the OPSR of VCSELs with a single transverse mode polarization decreases as the modulation speed increases. The drawback can be overcome by increasing the In content in GaInAs. In 2001, Nobuhiko Nishiyama et al. achieved the first GaInAs-based VCSEL with a wavelength of 1.13 μm on a GaAs(311)B substrate using the metal chemical vapor deposition technique, and the OPSR also exceeded 30 dB, and this laser performed well at high temperature characteristics [77]. In 2003, Yae L. Okuno used wafer bonding technology to integrate (113) InP-based active regions onto (001) GaAs-based DBRs to fabricate 1.3 μm VCSELs with a final achieved power extinction ratio of 31 dB [78]. In 2005, the effect of non-table structure on polarization control was investigated on the basis of wafer mismatch bonding, and it was demonstrated that 1.3 μm VCSELs can maintain polarization stability under high speed modulation at 1 Gb/s [79]. In addition to designing the active region as an asymmetric structure, the asymmetric current injection also allows for polarization control. The VCSEL cross-sections for symmetric current injection and asymmetric current injection are shown in Figure 9a,b. In 2000, G. Verschaffelt et al. designed an intracavity contact VCSEL based on an asymmetric contact layout and demonstrated that polarization is indeed affected by asymmetric current injection [80]. However, the asymmetric current injection affects the laser performance of the VCSEL, which is less effective in controlling the polarization.

### 4.3. Surface Grating

The reflectance and transmittance of a linear grating depend on the polarization of the incident electromagnetic wave, so combining a surface grating with a DBR yields a polarization-dependent reflector. Surface grating etching for polarization control requires only a few additional steps to the standard VCSEL process, and provides excellent polarization control. The initial solution for surface grating etching was to deposit a layer of metal on the side wall of the etched grating for polarization control [81]. However, the loss of metal grating is too large, and the manufacturing process is too complicated, which is not conducive to the increase of output power and large-scale application. This problem can be overcome by using a single integrated shallow surface grating. In 2004, Michalzik et al. etched a dielectric grating on the top laser surface to achieve polarization control and designed a VCSEL with an output power of 29 mW and an OPSR of 12 dB [82]. However, the disadvantage of the surface grating etching method is that it introduces diffraction loss, which increases the threshold current and reduces the output power. A better way is the inverted grating relief technique, which is to grow an extra quarter-wave layer on the standard VCSEL. The grating is etched in the center of the inverted layer, and the etching depth is controlled in the additional 1/4 wavelength thickness layer to reduce diffraction loss, and the threshold gain of the fundamental mode is significantly reduced while polarization control is performed. In 2005, Johannes Michael Ostermann et al. achieved stable polarization in both single-mode and multimode VCSELs using monolithic integrated inverted grating relief, with a single-mode output power of 4.3 mW and multimode output power of 10 mW [83]. In the same year, Johannes Michael Ostermann et al. compared the effects of polarization control of standard VCSELs and inverted grating relief VCSELs, where all surface relief VCSELs had stable polarization and 64% of polarization VCSELs had polarization switching. In addition, the inverted grating relief VCSELs deliver more than three times the maximum single-mode power compared to standard VCSELs [84]. Stress is inevitably introduced in the normal VCSEL fabrication process, so it is necessary to understand whether the polarization control method used can maintain stable polarization despite the presence of applied stress. In 2007, Johannes Michael Ostermann et al. tested whether surface gratings could still maintain stable polarization by mechanically bending the crystal plane to introduce external stresses [85]. The experimental results show that the surface grating method can perform polarization control even in the presence of stress, proving the reliability of the surface grating method for polarization control. In 2011, Ahmed Al-Samaneh et al. used inverted grating relief for polarization control. VCSEL is designed for MEMS atomic clock with OPSR of 21 dB at 65 °C and modulation bandwidth of 5GHz at a bias current of only 0.5 mA [60]. In 2012, Ahmed Al-Samaneh et al. implemented a single-mode, single-polarization VCSEL for atomic clocks using inverted grating etching, achieving an SMSR of 20 dB at a 5 μm oxide aperture and an OPSR better than 20 dB at substrate temperatures above 100 °C [61]. In 2013, Alhmed Al-Samaneh further investigated the polarization control of VCSELs at high temperatures and designed VCSELs with a peak-to-peak difference of 25 dB between the main and suppressed polarization modes even at substrate temperatures above 80 °C [62]. In 2018, Y.Y. Li et al. improved the large-scale grating VCSEL fabrication process and fabricated an 894-nm VCSEL for application in atomic clocks with an OPSR of 30 dB at temperatures above 80 °C [58]. Research progress of the single-polarization control method of VCSEL is shown in Table 2.

### 4.4. Current Commercial VCSELs for Atomic Magnetometers

Optical communication and networks have been the main driver for the development of VCSELs since their commercial introduction. However, VCSELs for optical communications and networks are typically available at 850 nm, 1310 nm, or 1550 nm. With a chip-scale atomic magnetometer based on the optically pumped, the wavelength of VCSEL has to meet the requirements of atomic transitions to enable optical pumping (Cs at 852 or 895 nm or Rb at 780 or 795 nm). Furthermore, the application of atomic magnetometer requires the single-mode VCSEL to operate in a stable, linear state of polarization and to have low intensity and phase noise. Therefore, there are fewer manufacturers producing VCSELs for atomic sensing due to the difficulty of production and low demand. The current commercially available VCSELs for atomic magnetometers are listed in Table 3.

## 5. Application of VCSEL in a Chip-Scale Atomic Magnetometer

As mentioned above, chip-scale atomic magnetometers have gradually improved in sensitivity and have reached subfemtotesla magnitude [88,89], enabling sensitivity similar to that of SQUIDs, and without the need for ultra-low temperature environments. Generally, the light sources of choice are diode lasers, where distributed feedback (DFB) lasers are widely used because of their extremely narrow linewidth and high frequency stability. However, the volume of DFB lasers is too large for a chip-scale magnetometer. To solve this problem, the light source of the atomic magnetometer was replaced by an optical fiber instead of a spatial light. Nevertheless, because of the coupling efficiency, optical fiber leads to a loss of optical power and reduces the portability of the device. The method of using VCSELs as light sources not only makes it easy to integrate miniaturized prototypes and even achieve battery-powered portability, but also results in less loss of optical power. The following will introduce the latest progress of three different types of chip-scale atomic magnetometers.

### 5.1. CPT Atomic Magnetometer

**Figure 10:**
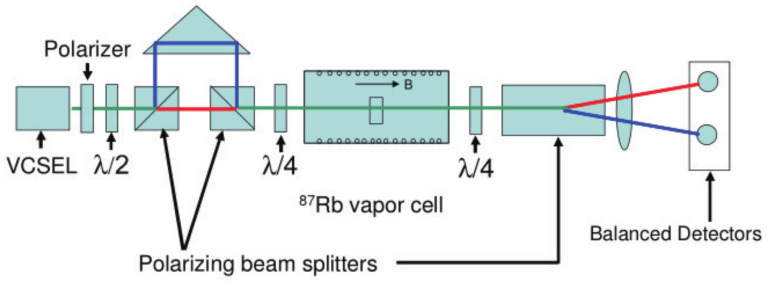
The experimental setup for differential detection [91].

CPT atomic magnetometers are generally used to measure cardiac magnetism because of its low sensitivity. The structure of CPT atomic magnetometer is similar to that of CPT atomic clock, and the technology of miniaturized CPT atomic clock is relatively mature, so miniaturized CPT atomic magnetometer is developing rapidly. In 2004, Peter D. D. Schwindt et al. fabricated a chip-scale atomic magnetometer based on the CPT resonance principle, in which the power of the VCSEL was attenuated to 5 μW and the beam diameter was 170 μm after collimation. The sensitivity of the device is 50 ${\mathrm{pT}/\mathrm{Hz}}^{1/2}$ and the volume is 12 ${\mathrm{mm}}^{3}$ [90]. To improve sensitivity of the miniaturization of the CPT atomic magnetometer, Michael Rosenbluh et al. suppressed the noise in the CPT resonance detection process by differential detection with a reduction of two orders of magnitude in the low-frequency noise power spectral density. This provides a new approach to the sensitivity enhancement of magnetometer [91]. The experimental setup for differential detection is shown in Figure 10. In 2006, Lammegger presented a coupled dark state magnetometer (CSDM), which was based on two-photon spectroscopy of free alkali atoms. The vertical cavity of VCSELs leads to the characteristic of low parasitic capacity and therefore can be modulated by microwave signals with good efficiency [92]. In 2010, Pollinger et al. designed a laser carrier frequency stabilization control loop for CSDM which had been proven in long-term magnetic field measurements up to 11 h [93]. Due to its high accuracy and stability, CSDM was used to detect a space magnetic field [94,95,96].

**Figure 11:**
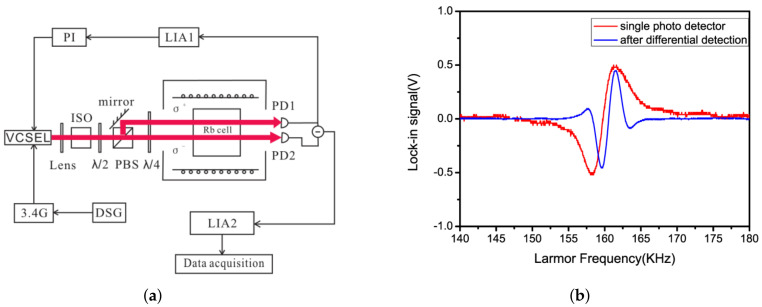
(**a**) The experimental setup for differential detection; (**b**) the lock-in signals after differential detection and of the single photo detector [102].

In 2010, Guobin Liu et al. improved the microwave modulation scheme of the CPT atomic clock by proposing an energy-level modulation scheme that extends the detection resolution of the magnetic field [97]. However, this scheme can reduce the CPT signal amplitude due to the uneven magnetic field of the AC coil. Generally, the circular polarization multichromatic laser was used to generate CPT resonance that causes the problems of leaky trap-state atoms and the unwanted light background. In order to improve the atom utilization, many new CPT configurations have been proposed, such as lin//lin [98], push–pull [99], $\sigma_{+}-\sigma_{-}$ [100], etc. In 2012, Tan et al. proposed a chip-level lin⊥lin quasi-bichromatic laser beam scheme in which two VCSELs are set up as a master–slave scheme. The master laser is driven by DC, and the slave laser is driven by a 6.834 GHz microwave [101]. Frequency injection locking is achieved by feeding output of the master circuit back to the slave circuit. In 2014, Shangqing Liang et al. proposed a new differential detection method for CPT magnetometers, which is able to improve both sensitivity and absolute accuracy [102]. The experimental setup is shown in Figure 11a. The comparison between the signal after differential detection and the signal without differential detection is shown in Figure 11b. Before entering the cell, two orthogonal beams of linearly polarized light are obtained by PBS beam splitting, and then left-rotating circularly polarized light and right-rotating circularly polarized light are obtained by passing through a quarter-wave plate.

In 2016, Haje Korth et al. fabricated a miniaturized scalar magnetometer for space exploration applications [9]. The VCSEL in the magnetometer is temperature and current controlled by a custom mixed-signal integrated circuit. The wavelength is modified by slowly scanning the temperature of VCSEL to find the temperature point when the ${}^{87}Rb$ D1 line is reached, and this temperature point is used as the set value for feedback control to achieve closed-loop control of the frequency. In 2022, V. Andryushkov et al. used a compensated modulation coil to convert a CPT atomic clock into a vector magnetometer [103]. The method allows for configuring the atomic magnetometer without changing the CPT atomic clock components. Despite the rapid miniaturization of CPT atomic magnetometers, they are gradually being replaced by other types of optical pumping magnetometers in the medical field due to their inherent low sensitivity.

### 5.2. M-x Atomic Magnetometer and Bell–Bloom Atomic Magnetometer

M-x atomic magnetometers use Helmholtz coils to coherently drive the precession of the atomic spin about the static magnetic field. The alkali atomic spin precession modulates the transmitted light intensity, which is collected by a photodetector. Unlike the M-x magnetometer, the Bell–Bloom magnetometer modulates the pump light, which drives the coherent precession of the alkali atoms about the external magnetic field to be measured. In 2006, S. Groeger designed a highly sensitive laser-pumped M-x magnetometer with higher magnetometric sensitivity for laser pumping compared to discharge lamp pumping. The light source used in the device is a frequency-stabilized laser introduced through a 10 m long fiber. Although the magnetometer was not miniaturized and integrated in this experiment, it was demonstrated that the sensitivity of the M-x magnetometer with the laser as the light source can reach 29 fT [31]. In 2007, Peter D. D. Schwindt et al. constructed the first miniature optically pumped atomic magnetometer, which reduces power consumption, simplifies operation, and achieves higher sensitivity than previous magnetometers based on the CPT resonance principle [104]. The laser used in the experiment is a 795 nm VCSEL, and the heating of the gas chamber and laser is performed by a double-layer coil magnetic field counteracting technique, which can greatly reduce the influence of the magnetic field brought by the heating current. The overall power consumption of the device is 194 mW, and the overall volume is 25 ${\mathrm{mm}}^{3}$. In 2010, in order to overcome the crosstalk problem in arraying the mid-drive coils of the M-x magnetometer, Ricardo Jimenez-Martinez et al. abandoned the scheme of using RF coils in favor of direct modulation of the VCSEL [105]. By adjusting the modulation amplitude and duty cycle in the Bell–Bloom magnetometer, a sensitivity similar to that of the M-x magnetometer was obtained. In 2017, M. Ranjbaran used square-wave RF magnetic fields with different duty cycles instead of sinusoidal fields to enhance the harmonic component [106]. It was found that the steepest slope of the dispersive signal was obtained for the detection of nuclear resonance at the 5th harmonic of the square wave field with a duty cycle of 10%, resulting in a 4.5-fold increase in magnetic field sensitivity relative to the first harmonic. Although this experiment uses a DFB laser, the results are also valid for chip-scale atomic magnetometers with VCSEL as the light source. In the Bell–Bloom magnetometer, modulating laser power reduces its linearity, and to address this issue, Levy et al. improved the linearity of the Bell–Bloom magnetometer by offsetting the large signal generated at the photodetector due to light not absorbed by the magnetometer [107]. Enabling the Bell–Bloom magnetometer to operate in magnetic interference-rich environments, it is suitable for measuring human biomagnetic fields in non-magnetically shielded environments. In 2018, Hunter et al. presented the application of a free-induction-decay (FID) technique using the amplitude modulation (AM) and frequency modulation (FM) implementations with a microfabricated Cs vapor cell. VCSEL was the solitary laser source for atomic magnetometer due to compact structure and large modulation bandwidth. However, due to the limitation of optical power, the noise floor of the root spectral density was measured to be 3 ${\mathrm{pT}/\mathrm{Hz}}^{1/2}$ and 16 ${\mathrm{pT}/\mathrm{Hz}}^{1/2}$ for the AM and FM configurations, respectively [108]. In the same year, Deans et al. reported on a single-channel rubidium radio-frequency (RF) atomic magnetometer operating in unshielded environments and near temperature with a measured sensitivity of 130 ${\mathrm{fT}/\mathrm{Hz}}^{1/2}$ [109]. The volume of sensor was only 57 ${\mathrm{mm}}^{3}$, which increases the spatial resolution of measurements. To demonstrate the feasibility of practical use of electromagnetic induction imaging with atomic magnetometers, Marmugi et al. reported a 50 ${\mathrm{fT}/\mathrm{Hz}}^{1/2}$ ${}^{87}Rb$ RF atomic magnetometer operating in an unshielded environment and near room temperature. Phase stability better than 0.03${}^{\circ }$ across the imaging area over several hours was obtained by biasing the RF atomic magnetometer to near-resonant operation [110]. In 2021, Deans et al. realized a compact RF atomic magnetometer with the sensor head including the required laser sources, the RF source as well as the magnetic field coils. VCSELs were tuned by varying the current and temperatures but do not have independent control of frequency and intensity. The advantage of this technique is that it does not require any background subtraction, which further validates its applicability to bio-medicine [111].

### 5.3. Dual-Beam SERF Atomic Magnetometer

The dual-beam SERF atomic magnetometer has a high sensitivity and is currently used in a wide range of applications in the field of brain magnetism. We summarized the recent advances in sensitivity enhancement of dual-beam SERF magnetometers. In 2003, I. K. Kominis et al. realized the first small volume multi-channel subfemtotesla atomic magnetometer based on SERF. Its sensitivity reached 0.54 ${\mathrm{fT}/\mathrm{Hz}}^{1/2}$, which exceeded that of superconducting quantum interference devices, and the measurement volume was only 0.3 ${\mathrm{cm}}^{3}$ [88]. The experiment uses a high power laser with a pump optical power of 1W and a detection optical power of 100 mW, and a single VCSEL cannot achieve such a high output power. In 2007, Vishal Shah et al. realized a single-beam SERF chip-scale atomic magnetometer with a sensitivity below 70 ${\mathrm{fT}/\mathrm{Hz}}^{1/2}$ [112]. The laser power of the sensor is only 0.35 mW for single-beam measurement and 0.12 mW for dual-beam measurement of the detection light, which can use VCSEL as the light source.

**Figure 12:**
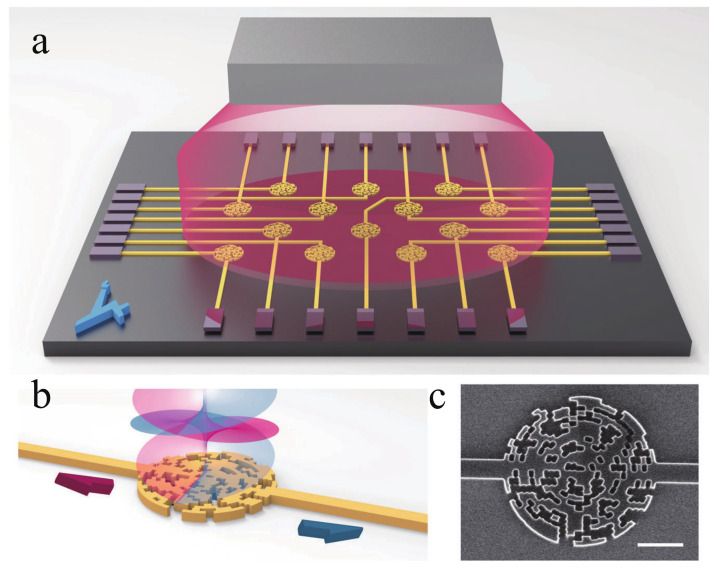
(**a**) Conceptual sketch of integrated magnetic sensor array; (**b**) schematic illustration showing the operation of spin selector; (**c**) scanning electron microscope(SEM) of the fabricated device [116].

Suppression of magnetic field noise can effectively improve the sensitivity of SERF magnetometers. An effective way is to use magnetically shielded materials with less magnetic noise, such as magnetic shielding using Mn-Zn ferrites. Another way is to minimize the magnetic field introduced by the current of the electronic components. In 2009, Jan Preusser et al. performed optical pumping and heating by moving electronic components such as VCSELs and heating membranes to a remote location and using optical fiber introduction, a method that effectively avoids the effects of magnetic noise generated by electric currents [113]. In 2013, R. Mhaskar et al. improved the laser heating of the gas chamber by using a filter attached to the chamber wall to absorb the laser light for heating, reducing the reflection and refraction from the chamber wall and reducing the power required for laser heating [114]. With the development of integrated optoelectronics, great breakthroughs have been made in nano-chip atomic magnetometers. In 2020, Yoel Sebbag et al. designed a chip-scale nanophotonic atomic magnetic sensor. The magnetic field detection is performed by measuring the change in ellipticity due to magnetically correlated circular dichroism by means of a photon spin sorter (PSS) [115]. In 2021, Yoel Sebbag et al. implemented a new high-precision atomic magnetometer with a magnetic sensitivity of 700 ${\mathrm{pT}/\mathrm{Hz}}^{1/2}$ by the computer-aided reverse design of a photon spin sorter [116]. The two-dimensional mapping of the magnetic field of the integrated sensor array is shown in Figure 12, and the proposed photonic chip atomic magnetometer offers a new solution to reduce the size. In 2022, Guiying Zhang et al. integrated a miniaturized atomic magnetometer using a VCSEL as the light source. The experiment used a single beam scheme, but the incident beam was changed from circularly polarized to elliptically polarized [117]. The circularly polarized component of elliptically polarized light is used for optical pumping, and the linearly polarized component is used for spin detection, which in turn suppresses magnetometer noise. The magnetometer has a sensitivity of 35 ${\mathrm{fT}/\mathrm{Hz}}^{1/2}$ and can be used for cardiac magnetic measurements. Some recent applications for SERF magnetometers related to parameter optimization [118], differential measurement [119,120], and triaxial measurement [121] are using DFB lasers for the light source, and perhaps VCSELs with low noise and high frequency stability will be used as the light source for further miniaturization and integration.

## 6. Atomic Magnetometer in the Medical Field

### 6.1. MCG Measurements with Chip-Scale Atomic Magnetometer

**Figure 13:**
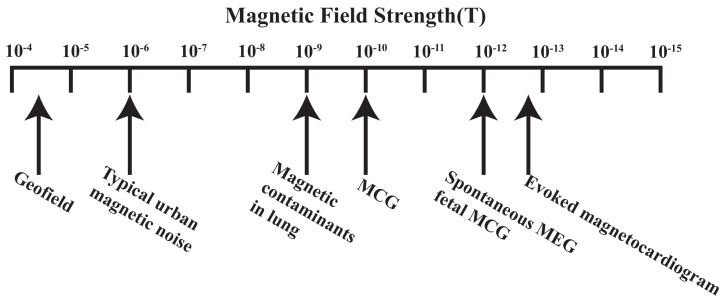
Amplitude of various biomagnetism signals in comparison to the geofield and urban magnetic noise [123].

The magnetic field produced by the human heart carries valuable information for medical research, as well as for diagnostics and screening for disease [122]. Nowadays, the sensitivity of SERF magnetometer has achieved 0.54 ${\mathrm{fT}/\mathrm{Hz}}^{1/2}$ under laboratory conditions [88]. In addition, the sensitivity of chip-scale atomic magnetometer has achieved 70 ${\mathrm{fT}/\mathrm{Hz}}^{1/2}$ [112]. As can be seen in Figure 13, the SERF magnetometer has sufficient sensitivity to measure MEG and MCG signals.

**Figure 14:**
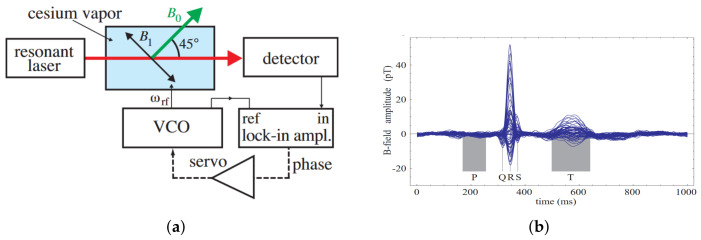
(**a**) Schematic set up of the optically-pumped Mx-magnetometer in the phase-locked mode; (**b**) MCGs taken on the grid above the chest, where P indicates atrial depolarization, QRS is responsible for ventricular depolarization, and T represents ventricular repolarization [122].

Unlike electric signals, which are measured with ECGs and electroencephalograms (EEGs), magnetic signals are not affected by the biological tissues because of the nearly homogeneous permeability of the human body, making it more reliable for the detection of biological phenomena [124]. Therefore, ECG and MCG give the opportunity to better diagnose cardiac arrhythmia and coronary artery diseases. In the last two decades, in order to improve the spatial resolution, the structure of the SERF magnetometer measuring MCG signals has been changed from single channel to multi-channel. In 2003, Bison et al. used an optically-pumped Mx magnetometer to produce movies of the temporal dynamics of the human cardiomagnetic field map, and compared with signals generated by SQUID magnetometers. The MCG measured by optically-pumped Mx magnetometer was experimentally shown to be valid. Figure 14a,b show the structure used and the measured PQRST signal.

**Figure 15:**
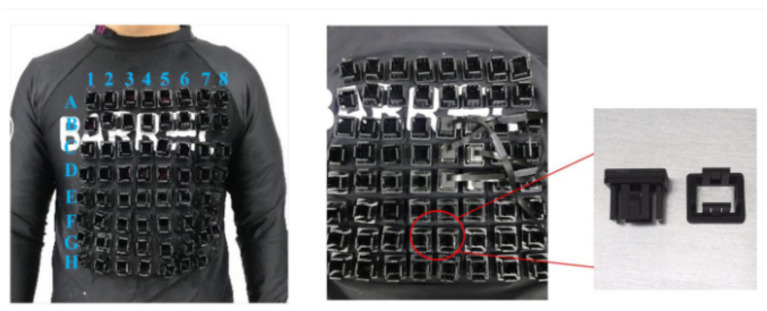
Position labels of the 8 × 8 array with 30 mm intervals, QZFM magnetometer and customized receptacles [127].

In 2018, Kim et al. implemented a 16-channel atomic magnetometer using a flat structured rubidium gas cell with a sensitivity of several tens of femtotesla at low frequencies. With the improvement of chip-scale atomic magnetometer sensitivity and size reduction, it is gradually applied to measure the MCG signal of the fetus. The chip-scale atomic magnetometer can be placed in variable geometries and is particularly suitable for flexible multichannel fMCG arrays, since the curvature of the abdomen during pregnancy is difficult to cover with large sensors or static sensor array [125]. The noninvasively monitored ECG signals are generally of poor quality because the electrical potential, conducted from the fetal heart to the maternal abdomen, are attenuated by the insulating waxy layer around the skin called vernix caseosa, which protects the skin of the fetus from the aqueous environment of the womb [126]. Therefore, fetal MCG has a greater signal intensity compared to fetal ECG. Another trend in the development of chip-scale atomic magnetometer is the move to wearable devices. In 2021, yanfeiyang et al. developed a wearable multichannel MCG system based on a SERF atomic magnetometer array. The magnetometer used in the experiment is QZFM, which uses a VCSEL light source [127]. Figure 15 shows the array of magnetometers worn by the subjects.

### 6.2. MEG Measurements with a Chip-Scale Atomic Magnetometer

**Figure 16:**
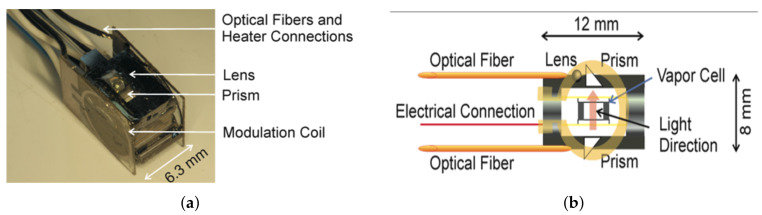
(**a**) Physical packaging of magnetometer; (**b**) magnetometer structure schematic [128].

In 2012, T. H. Sander et al. used a chip-scale atomic magnetometer for the first time to measure somatosensory evoked and spontaneous magnetic brain signals with sensor probe images shown in Figure 16 [128].

**Figure 17:**
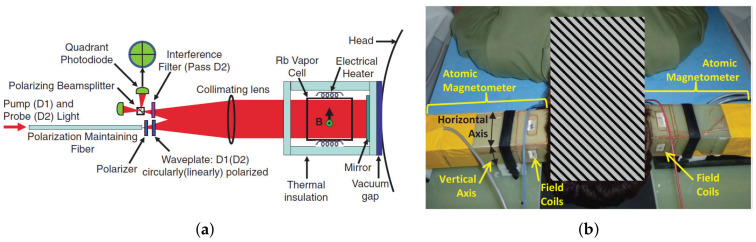
(**a**) Physical packaging of magnetometer; (**b**) experimental map of the brain magnetometer [129].

In 2013, Cort N Johnson et al. demonstrated the first multi-sensor magnetoencephalography. The magnetometer structure and the magnetic brain measurement experiment are shown in Figure 17a,b, respectively. To reduce the effects caused by external magnetic fields and to maximize the brain magnetic signal, the magnetometer uses a fiber optic introduction and a reflective optical path [129].

**Figure 18:**
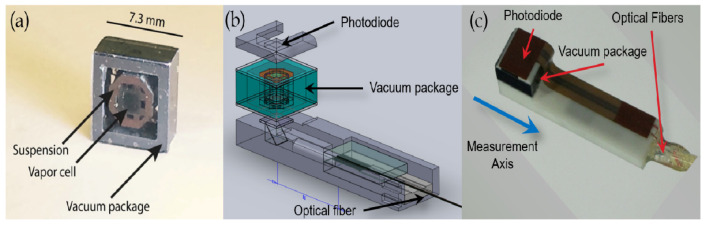
A fiber-optically coupled chip-scale atomic magnetometer. (**a**) the vacuum assembly housing the vapor cell; (**b**) a schematic of the optical bench; (**c**) a photograph of the final package [8].

In 2014, Orang Alem et al. used a miniature magnetometer to measure the magnetic brain signal in epileptic mice, which has important implications for the subsequent treatment of patients with epilepsy [7]. In 2017, Orang Alem et al. further reduced the magnetometer probe and constructed a multichannel imaging system consisting of 25 optically pumped magnetometers with a sensitive volume of 1.5 cm × 1.5 cm × 1.5 cm sensors which are introduced through two optical fibers, one beam for heating the gas chamber and laser, and the other beam for optical pumping [8]. The sensor has an average magnetic sensitivity of 24 fT when operating in a zero-magnetic environment and 5 ${\mathrm{pT}/\mathrm{Hz}}^{1/2}$ when operating in a geomagnetic environment. Figure 18 shows the structural diagram and vacuum package diagram of the magnetometer probe.

**Figure 19:**
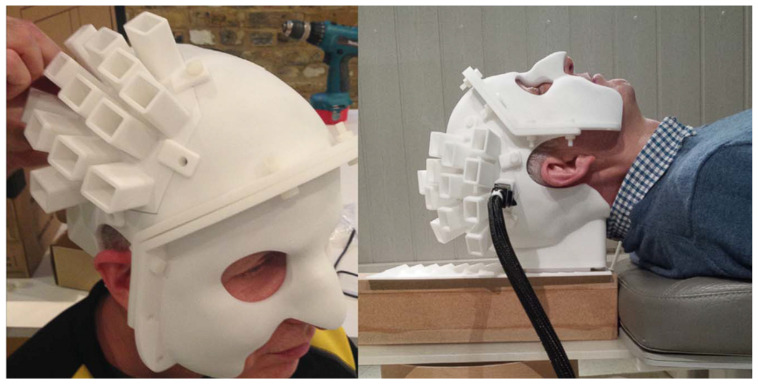
The 3D printed head model used for the experiments [130].

In the same year, Elena Boto et al. used a 3D printed head model to precisely sense the position and orientation of the sensor, substantially improving the signal-to-noise ratio of the light-pumped magnetometer. This approach allowed for more accurate imaging of magnetoencephalography. In addition, an additional reference sensor was added to reduce the effect of environmental interference. The experimentally measured signal amplitude is about four times that of the SQUID [130]. The 3D printed head model used for the experiments is shown in Figure 19.

In 2020, N.V. Nardelli et al. used a 24-channel array of first-order gradiometers in which 48 micromachined SERF magnetometers [131] were operated simultaneously for brain magnetometry. The magnetometer was operated at the zero field by an active magnetically shielded coil, and the gradiometers were rationally arranged to suppress laser- and fiber-induced noise. The median sensitivity of the magnetometer array reached 15.4 ${\mathrm{fT}/\mathrm{Hz}}^{1/2}$ [132]. In 2021, Jin Zhang et al. designed the SERF atomic magnetometer with a spherical gas chamber for cardio magnetography measurements. Under magnetically shielded conditions, the magnetometer sensitivity reached 125 ${\mathrm{fT}/\mathrm{Hz}}^{1/2}$ at 15 Hz and was able to obtain morphologically clear cardio magnetic signals [133]. The main competitor of VCSELs in the application of bio-sensing is the DFB laser, which has the advantages of high power, narrow line-width, low noise, and high stability. The approach of DFB lasers with fiber-optic in the atomic magnetometer can effectively reduce the magnetic field noise caused by the current. However, DFB lasers have the disadvantages of large volume and high costs, which makes the DFB-based atomic magnetometer unsuitable for industrial production. VCSELs have advantages in volume and cost, and the current commercial chip-scale magnetometers use VCSEL as the light source. Research progress of an optically pumped magnetometer in the field of magnetocardiography (MCG) and magnetoencephalography (MEG) is shown in Table 4.

## 7. Conclusions

With concept originating in the 1970s, VCSEL experienced rapid development in the past several decades. Meanwhile, the development of VCSEL-integrated atomic biosensors in recent years has opened a new era for this device mainly due to its compatibility with nano fabrication, which facilitates the manufacture of chip-scale atomic bio-sensing devices. Nowadays, VCSELs designed for chip-scale atomic devices are moving from the laboratory to various medical applications, which is attracting extensive attention in both academia and industry.

In this review, we summarized and discussed the recent development of VCSEL based chip-scale atomic biosensors, especially biosensing atomic magnetometers, which is undergoing rapid development due to its outstanding capabilities in mapping human generated magnetic fields. Although it is acknowledged that atomic magnetometers of this kind may replace traditional SQUID systems in the near future for various medical applications, challenges still exist including mass multi-sensor alignment and high sensitive SERF magnetometers (with sensitivity down to femtotesla); in addition, the current and heat of VCSEL near alkali vapor cell may couple with the electro-heat-magnetic system (of the vapor cell). Studies have been done in which fiber-optic approach is applied as a light source, which ends up with similar sensitivity while introducing the bulk DFB laser system [149]. Nevertheless, based on published knowledge, VCSEL integration in atomic magnetometers as light sources showed outstanding advantages in terms of portability, industrialization, and cost performance (over 90% reduction compared to the fiber-optic approach). In addition, emerging technologies are promoting the performance of VCSELs of this kind, such as the integration of metasurfaces that enables further reduction in size with wavefront control, improvement of noise suppression methods in pursuit of improved sensitivity, novel CMOS compatible techniques that facilitate mass production, etc. In conventional practice, the most economical and efficient way to control the mode and polarization of VCSELs (in biosensing magnetometers) is surface grating etching, while emerging studies bring attractive features to VCSELs, namely integrated polarization splitting or integration [120] with metalens [150,151].

Driven by the increasing demand of medical-sensing and medical-monitor industry, VCSEL integration in bio-sensing atomic devices is drawing extensive attention both in academia and industry. In addition, with the development of integrated photonics and CMOS compatible nano fabrication technology, VCSEL integration of this kind is undergoing rapid development. It is promising that bio-sensing atomic devices integrated with VCSEL may experience development in the near future with related application marching to the medical instrumentation market. Currently, it seems that the most economical and efficient way to control the mode and polarization of VCSELs is surface grating etching, but some new structures may bring some attractive features to VCSELs. VCSELs applied to chip-scale atomic devices have moved from the laboratory to the market, which have made an important contribution to the reduction of device size and power consumption.

## Figures and Tables

**Table 1:** Research progress of single-mode control method of VCSEL.

Year	Wavelength (nm)	Oxide Aperture (μm)	Output Power (mW)	Method	Ref
1997	850	3	2.8	Small oxide aperture	[35]
2000	940	7	5	Extended cavity	[37]
2001	980	9.2	5.4	Extended cavity	[34]
2004	850	8	3.8	Ion implantation	[39]
2006	850	4	4.7	Metal aperture	[43]
2007	850	10 × 10	1	High-contrast subwavelength grating	[55]
2008	794.7	\	0.8	\	[59]
2008	850	10	2.3	High-contrast subwavelength grating	[56]
2010	1310	11	0.4	High-contrast subwavelength grating	[27]
2011	894.6	3	0.8	Inverted grating relief	[60]
2012	894.6	5	1.5	Inverted grating relief	[61]
2013	894.6	4	0.8	Inverted grating relief	[62]
2016	850	3.75	3	Dielectric anti-phase filter	[45]
2017	894.6	7	0.45	Surface relief	[46]
2019	1300	\	13.3	Photonic crystal	[53]
2022	795	6	4.1	Increase the distance between active layer and aperture	[63]
2022	940	4 × 8	0.8	High-contrast subwavelength grating	[57]

**Table 2:** Research progress of the single-polarization control method of VCSEL.

Year	Wavelength	Oxide Aperture	SMSR	OPSR	Method	Ref.
2011	894.6 nm	3–5 μm	50 dB	21 dB	Inverted grating	[60]
2012	894.6 nm	5 μm	20 dB	20 dB	Inverted grating	[61]
2013	894.6 nm	3–4 μm	42 dB	21 dB	Inverted grating	[62]
2015	843 nm	10 μm	30 dB	20 dB	elliptical photonic crystal	[71]
2018	894 nm	3–5 μm	\	30dB	Surface grating	[86]
2018	894 nm	$2\times 4.6\;\mu m^{2}$	25 dB	22 dB	eye-type oxide aperture	[73]
2021	894.6 nm	$2\times 4.6\;\mu m^{2}$	30 dB	25 dB	anisotropic oxidation apertures	[87]
2022	850 nm	$5\times 45\;\mu m^{2}$	\	15 dB	Anisotropic loss	[69]

**Table 3:** Current commercial VCSELs for atomic magnetometers.

Wavelength	Spectral Linewidth	Output Power	Operation Temperature	Threshold Current	Divergence Angle	SMSR	Package	Manufacture
795 nm	100 MHz	0.5 mW	Min: −20 ${}^{\circ }$C Max: 110 ${}^{\circ }$C	0.75 mA	Min: 15${}^{\circ }$ Max: 25${}^{\circ }$	20 dB	TO46	Vixar Massachusetts, USA
895 nm	50 MHz	0.5 mW	Min: −20 ${}^{\circ }$C Max: 110 ${}^{\circ }$C	0.5 mA	Min: 16${}^{\circ }$ Max: 26${}^{\circ }$	20 dB	TO46	Vixar Massachusetts, USA
795 nm	60 MHz	0.4 mW	Min: −10 ${}^{\circ }$C Max: 55 ${}^{\circ }$C	0.8 mA	Min: 10${}^{\circ }$ Max: 25${}^{\circ }$	20 dB	TO46,TO5	Ace photonics Jilin Province, China
795 nm	\	0.4 mW	Min: −20 ${}^{\circ }$C Max: 110 ${}^{\circ }$C	0.5 mA	Min: 18${}^{\circ }$ Max: 23${}^{\circ }$	\	TO46	Throlabs New Jersey, USA
895 nm	\	0.3 mW	Min: −20 ${}^{\circ }$C Max: 110 ${}^{\circ }$C	0.5 mA	Min: 16${}^{\circ }$ Max: 26${}^{\circ }$	\	TO46	Throlabs New Jersey, USA
780 nm	100 MHz	0.25 mW	Min: −20 ${}^{\circ }$C Max: 80 ${}^{\circ }$C	0.5 mA	Min: 10${}^{\circ }$ Max: 20${}^{\circ }$	30 dB	TO46	ULM Photonics Ulm, Germany
795 nm	100 MHz	0.25 mW	Min: −20 ${}^{\circ }$C Max: 80 ${}^{\circ }$C	0.5 mA	Min: 10${}^{\circ }$ Max: 25${}^{\circ }$	30 dB	TO46	ULM Photonics Ulm, Germany
850 nm	\	2.5 mW	Min: −40 ${}^{\circ }$C Max:85 ${}^{\circ }$C	2 mA	Min: 10${}^{\circ }$ Max: 20${}^{\circ }$	10 dB	TO46	PHILIPS Amsterdam, The Netherlands

**Table 4:** Research progress of an optically pumped magnetometer in the field of MCG and MEG.

Year	MEG or MCG	Number of Channels	3d B Bandwidths	Light Source	Size of Vapor Cell	Magnetometer Sensitivity	Ref
2006	MEG	6	\	DFB+Fiber	$7.5\times 7.5\times 7.5\;{\mathrm{cm}}^{3}$	$3.5\;{\mathrm{fT}/\mathrm{Hz}}^{1/2}$	[134]
2009	MCG	19	\	DFB+Fiber	R=30 mm	\	[135]
2010	MEG	4	20 Hz	DFB+Fiber	$2.5\times 2.5\times 2.5\;{\mathrm{cm}}^{3}$	$5\;{\mathrm{fT}/\mathrm{Hz}}^{1/2}$	[136]
2012	MEG	1	50 Hz	DFB+Fiber	$2\times 2\times 2\;{\mathrm{mm}}^{3}$	$200\;{\mathrm{fT}/\mathrm{Hz}}^{1/2}$	[137]
2012	MCG	4	\	DFB+Fiber	$1\times 1\times 5\;{\mathrm{cm}}^{3}$	$6–11\;{\mathrm{fT}/\mathrm{Hz}}^{1/2}$	[137]
2013	MEG	8	\	DFB+Fiber	$2.5\times 2.5\times 2.5\;{\mathrm{cm}}^{3}$	$5\;{\mathrm{fT}/\mathrm{Hz}}^{1/2}$	[129]
2013	MEG and MCG	1	100 Hz	DFB+Fiber	$2\times 2\times 5\;{\mathrm{cm}}^{3}$	$10\;{\mathrm{fT}/\mathrm{Hz}}^{1/2}$	[125]
2014	MEG	4	250 Hz	DFB+Fiber	$2\;{\mathrm{mm}}^{3}$	$30\;{\mathrm{fT}/\mathrm{Hz}}^{1/2}$	[7]
2014	MEG	256	≈190 Hz	DFB	$12\times 12\times 12\;{\mathrm{cm}}^{3}$	$4\;{\mathrm{fT}/\mathrm{Hz}}^{1/2}$	[2]
2016	MEG	4	90 Hz	DFB+Fiber	$2.5\times 2.5\times 0.4\;{\mathrm{cm}}^{3}$	$5\;{\mathrm{fT}/\mathrm{Hz}}^{1/2}$	[138]
2017	MEG	20	78∼95 Hz	DFB+Fiber	$2.5\times 2.5\times 0.4\;{\mathrm{cm}}^{3}$	$5\;{\mathrm{fT}/\mathrm{Hz}}^{1/2}$	[139]
2017	MEG	13	100 Hz	VCSEL	$3\times 3\times 3\;{\mathrm{mm}}^{3}$	$26.2\;{\mathrm{fT}/\mathrm{Hz}}^{1/2}$	[130]
2018	MEG	13	100 Hz	VCSEL	$3\times 3\times 3\;{\mathrm{mm}}^{3}$	$15\;{\mathrm{fT}/\mathrm{Hz}}^{1/2}$	[140]
2019	MEG	2	300 Hz	\	$1\times 1\times 1\;{\mathrm{cm}}^{3}$	$210\;{\mathrm{fT}/\mathrm{Hz}}^{1/2}$	[141]
2020	MCG	1	200 Hz	VCSEL	$3\times 3\times 3\;{\mathrm{mm}}^{3}$	$1\;{\mathrm{pT}/\mathrm{Hz}}^{1/2}$	[142]
2020	MEG	49	100 Hz	VCSEL	$3\times 3\times 3\;{\mathrm{mm}}^{3}$	$15\;{\mathrm{fT}/\mathrm{Hz}}^{1/2}$	[143]
2021	MEG	\	100 Hz	\	$3\times 4.5\times 5\;{\mathrm{mm}}^{3}$	$10\;{\mathrm{fT}/\mathrm{Hz}}^{1/2}$	[144]
2021	MEG	62	100 Hz	VCSEL	$3\times 3\times 3\;{\mathrm{mm}}^{3}$	$15\;{\mathrm{fT}/\mathrm{Hz}}^{1/2}$	[145]
2022	MEG	86	100 Hz	VCSEL	$3\times 3\times 3\;{\mathrm{mm}}^{3}$	$15\;{\mathrm{fT}/\mathrm{Hz}}^{1/2}$	[146,147]
2022	MEG	16	90 Hz	DFB+Fiber	$2.5\times 2.5\times 0.4\;{\mathrm{cm}}^{3}$	$5\;{\mathrm{fT}/\mathrm{Hz}}^{1/2}$	[148]

## Data Availability

Not applicable.
